# An Improved *Agrobacterium*-Mediated Transformation Method for an Important Fresh Fruit: Kiwifruit (*Actinidia deliciosa*)

**DOI:** 10.3390/plants14152353

**Published:** 2025-07-31

**Authors:** Chun-Lan Piao, Mengdou Ding, Yongbin Gao, Tao Song, Ying Zhu, Min-Long Cui

**Affiliations:** 1 Key Laboratory of Quality and Safety Control for Subtropical Fruit and Vegetable, Ministry of Agriculture and Rural Affairs, College of Horticulture Science, Zhejiang A&F University, Hangzhou 311300, China; 2 State Key Laboratory for Managing Biotic and Chemical Threats to the Quality and Safety of Agro-Products, Key Laboratory of Traceability for Agricultural Genetically Modified Organisms, Ministry of Agriculture and Rural Affairs, Institute of Virology and Biotechnology, Zhejiang Academy of Agricultural Sciences, Hangzhou 310021, China

**Keywords:** kiwifruit (*Actinidia deliciosa*), leaf explant, high-efficiency genetic transformation, Southern blot analysis, mature plant, stable *GFP* expression

## Abstract

Genetic transformation is an essential tool for investigating gene function and editing genomes. Kiwifruit, recognized as a significant global fresh fruit crop, holds considerable economic and nutritional importance. However, current genetic transformation techniques for kiwifruit are impeded by low efficiency, lengthy culture durations (a minimum of six months), and substantial labor requirements. In this research, we established an efficient system for shoot regeneration and the stable genetic transformation of the ‘Hayward’ cultivar, utilizing leaf explants in conjunction with two strains of *Agrobacterium* that harbor the expression vector pBI121-35S::GFP, which contains the green fluorescent protein (*GFP*) gene as a visible marker within the T-DNA region. Our results show that 93.3% of leaf explants responded positively to the regeneration medium, producing multiple independent adventitious shoots around the explants within a six-week period. Furthermore, over 71% of kanamycin-resistant plantlets exhibited robust *GFP* expression, and the entire transformation process was completed within four months of culture. Southern blot analysis confirmed the stable integration of *GFP* into the genome, while RT-PCR and fluorescence microscopy validated the sustained expression of *GFP* in mature plants. This efficient protocol for regeneration and transformation provides a solid foundation for micropropagation and the enhancement of desirable traits in kiwifruit through overexpression and gene silencing techniques.

## 1. Introduction

Kiwifruit (*Actinidia deliciosa*) is a globally important fresh fruit crop for economic and human health [1,2,3,4]. It contains many bioactive compounds such as rich vitamins, soluble sucrose, and flavonoids, and it has antioxidant activity [5,6,7,8,9,10]. The ability of classical breeding to improve the agronomic traits of kiwifruit such as maturity, disease resistance, and nutritional composition has been challenged, and progress has been slow due to the lack of useful genetic resources and longer breeding periods [11,12,13,14]. In contrast with traditional breeding, genetic transformation is a powerful research tool for altering secondary metabolic pathways to improve important traits in fruit through short-term application in gene discovery [15]. Interestingly, recently achieved results in the genomics, transcriptomics, and metabolomics of kiwifruit offer novel insights into the fundamental study of gene function and the regulatory network of important traits, further unraveling pathways for secondary biosynthesis and providing new ideas for new improved kiwifruit cultivars [13,16,17,18].

Genetic transformation is a fundamental tool not only for the development of new cultivars but also for the modulation of gene expression by overexpressing or knocking down genes such as through RNA interference and the CRISPR–Cas9 system [19,20,21,22]. Plant transformation has been accomplished using several techniques including particle gun and *Agrobacterium*-mediated methods [23,24,25,26]. Among these, *Agrobacterium*-mediated genetic transformation is the most widely used method for crop improvement and gene function analysis in plants [20,23,27]. Since the *Agrobacterium*-mediated transformation of kiwifruit from hypocotyls and stems was first reported by Uematsu et al. (1991) [23], several studies have reported *Agrobacterium*-mediated transformation methods from stems or leaves [28,29,30], but these methods present certain limitations such as low transformation efficiency, with successful transformants obtained through lengthy tissue culture periods and labor-intensive processes. Recently, it was also reported that *Agrobacterium rhizogenes* mediated transformation [31]. However, *Agrobacterium rhizogenes*-transformed shoots often exhibit altered morphology, including dwarfism, a decrease in apical dominance, an increase in the number of flowers, and abnormal root production [32].

In this study, we describe a protocol for stable and efficient plant regeneration and the *Agrobacterium*-mediated genetic transformation of kiwifruit using leaf explants. This stable and efficient plant regeneration and genetic transformation protocol provides useful tools for gene function analysis, micropropagation, and genome editing approaches to the genetic improvement of kiwifruit.

## 2. Results

### 2.1. Shoot Regeneration of Leaf Explants

**Figure 1:**
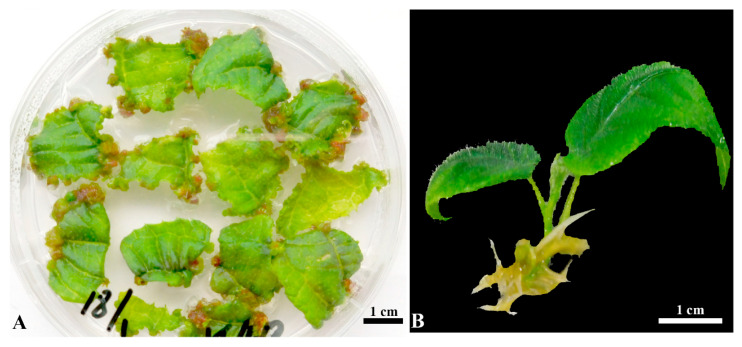
Plant regeneration from leaf explants of kiwifruit. (**A**) Shoot induction from leaf explants on 3/4 MS+ 5 mg/L BA + 1 mg/L Zt + 0.15 mg/L IBA medium (SIM) after 4 weeks of culture. (**B**) Root induction from regenerated shoots on 1/2 MS+ 0.25 mg/L IBA medium (RIM) after 6 weeks of culture.

To establish an efficient and renewable shoot regeneration system for kiwifruit cultivar ‘Hayward’, thirty leaf explants as a set were cultured on 3/4 MS basal medium supplemented with various concentrations and combinations of growth hormones (Table S2). After 10 days of culture, small swellings appeared at the leaf margins, and small adventitious shoots emerged in media containing high concentrations of BA alone or in combination with 1 mg/L Zt after four weeks of culture. Among these, more than 93% of the leaf explants responded to 3/4 MS medium supplemented with 5 mg/L BAP + 1 mg/L Zt + 0.15 mg/L IBA (SIM) and developed normal adventitious shoots (Figure 1A; Table S2). After 6 weeks of culture on SIM (Table S1), normally elongated shoots were excised and transferred to RIM (Table S1)-induced roots. More than 90% of shoots grew normally and successfully formed roots within 6 weeks of culture on rooting medium (Figure 1B). Therefore, SIM was chosen as the shoot induction medium for further transformation studies.

### 2.2. Transformation of Kiwifruit

**Figure 2:**
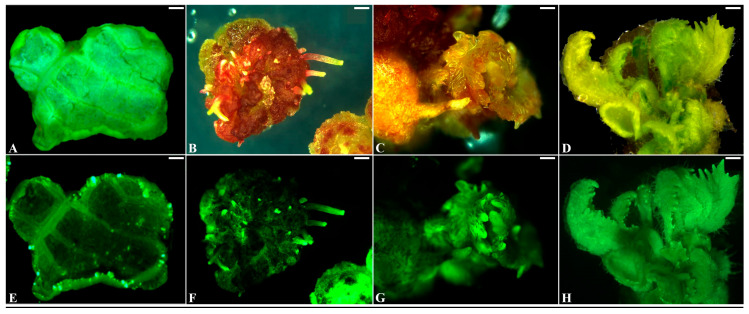
Observation of GFP expression at different steps in *Agrobacterium*-mediated transformation of kiwifruit. (**A**) Kanamycin-resistant small swellings emerged on wounded leaf segments on solid SM after 10 days of culture. (**B**) Protuberance formation on kanamycin-resistant swellings on solid SM after 3 weeks of culture. (**C**) Kanamycin-resistant small shoots emerged on solid SM after 5 weeks of culture. (**D**) Normally grown kanamycin-resistant shoots on solid SM after 8 weeks of culture. (**E**) GFP observation in kanamycin-resistant small swellings (**A**). (**F**) GFP observation in kanamycin-resistant protuberances (**B**). (**G**) GFP observation in kanamycin-resistant small shoots (**C**). (**H**) GFP observation in kanamycin-resistant plantlets (**D**). Bar = 1 mm.

**Figure 3:**
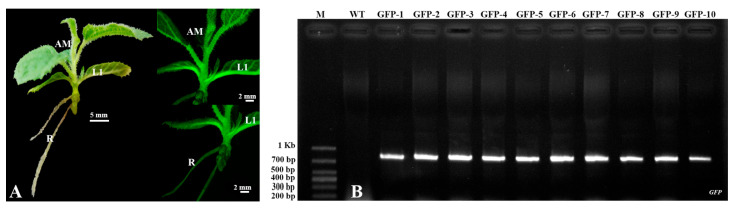
PCR analysis of transformed kiwifruits. Genomic DNA was extracted from fresh leaves of pot-grown wild-type (WT) plants and ten putative GFP-positive transgenic kiwifruit lines. (**A**) Rooted transgenic kiwifruit plant exhibiting GFP fluorescence after 6 weeks of culture on RIM supplemented with 50 mg/L kanamycin. (**B**) PCR detection of *GFP* transgene in ten independent transgenic lines using gene-specific primers (Table S3). WT: Non-transformed wild-type kiwifruit. GFP1–5: Five independent GFP-positive kiwifruits transformed with EHA105/pBI-35S::GFP. GFP6–10: Five independent GFP-positive kiwifruits transformed with GV3101/pBI-35S::GFP. M: DL1000 DNA marker (Takara, Japan). R: root; L1: First true leaf; AM: Apical meristem.

To establish an efficient *Agrobacterium*-mediated genetic transformation system for kiwifruit, we evaluated two common *Agrobacterium* strains, EHA105/pBI-35S::GFP and GV3101/pBI-35S::GFP, for their ability to transform leaf explants. The leaf explants were inoculated with diluted *Agrobacterium* for 15 min, followed by cocultivation for 3 days on CM (Table S2) at 25 °C in the dark and then being transferred to SM (Table S2). The transformation was repeated twice (Table 1). After 10 days of culture on SM, many small swells exhibiting strong *GFP* expression formed surrounding the leaf explants (Figure 2A,E). After 3 weeks of culture, protuberances with strong *GFP* expression appeared on many swellings (Figure 2B,F), and adventitious shoots developed from swellings after 5 weeks of culture on SM (Figure 2C,G). After 8 weeks of culture on SM, some normally growing adventitious shoots (Figure 2D,H) were excised and transferred to solid RIM (Table S1) supplemented with 50 mg/L kanamycin to induced roots. Over 71% of the adventitious shoots grew normally, formed roots, and presented strong *GFP* expression within 7 weeks of culture on RIM (Figure 3A; Table 1).

### 2.3. PCR Detection of Transgenic Kiwifruit

*GFP* expression was detected in the leaves, stems, and roots of small, regenerated kiwifruit plants after 6 weeks of culture on RIM (Table S1) supplemented with 50 mg/L kanamycin and 300 mg/L cefotaxime (Figure 3A). To confirm the presence of *GFP* and the kanamycin resistance gene *NPT II* in the kiwifruit genome, we used genomic DNA extracted from putatively transformed plants that survived kanamycin treatment and observed *GFP* expression with PCR amplification analysis. The results showed that the 780 bp band of the *GFP* gene and the 450 bp band of the *NPT II* gene were amplified in all investigated transformed kiwifruits (Figure 3B and Figure S1). However, no band was detected in the non-transformed wild-type (WT) kiwifruit plants (Figure 3B and Figure S1).

### 2.4. Southern Blot Analysis of Transgenic Kiwifruit

**Figure 4:**
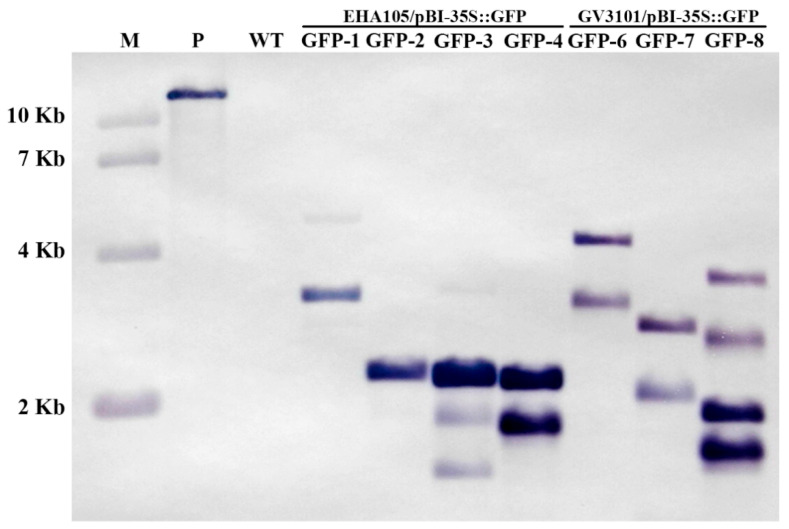
A Southern blot analysis of *GFP* integration in transformed kiwifruits. DNA from a non-transformed plant and seven GFP-positive transgenic kiwifruits was digested with *Bam*HI, fractionated using electrophoresis, transferred to a nylon membrane, and hybridized with the full-length *GFP* probe. WT: a non-transformed wild-type kiwifruit. GFP1–4: four independent transgenic kiwifruits transformed with EHA105/pBI-35S::GFP. GFP6–8: three independent transgenic kiwifruits transformed with GV3101/pBI-35S::GFP. M: DL 10,000 DNA marker (Takara, Japan). P: plasmid pBI-35S::GFP.

To confirm the integration of *GFP* in the kiwifruit genome, seven greenhouse-grown transgenic plants were analyzed using Southern hybridization with a probe containing the *GFP* fragment amplified with PCR from pBI-35S::GFP. The DNA from a wild-type (WT) plant grown under in vitro conditions was used as a negative control. Although all seven plants presented strongly different patterns of hybridization signals, hybridization signals were not detected in the WT plants (Figure 4), providing strong evidence that integrated copies of *GFP* sequences were present in the genomes of the transgenic plants (Figure 4).

### 2.5. Observation of GFP Expression in Potted Kiwifruit

**Figure 5:**
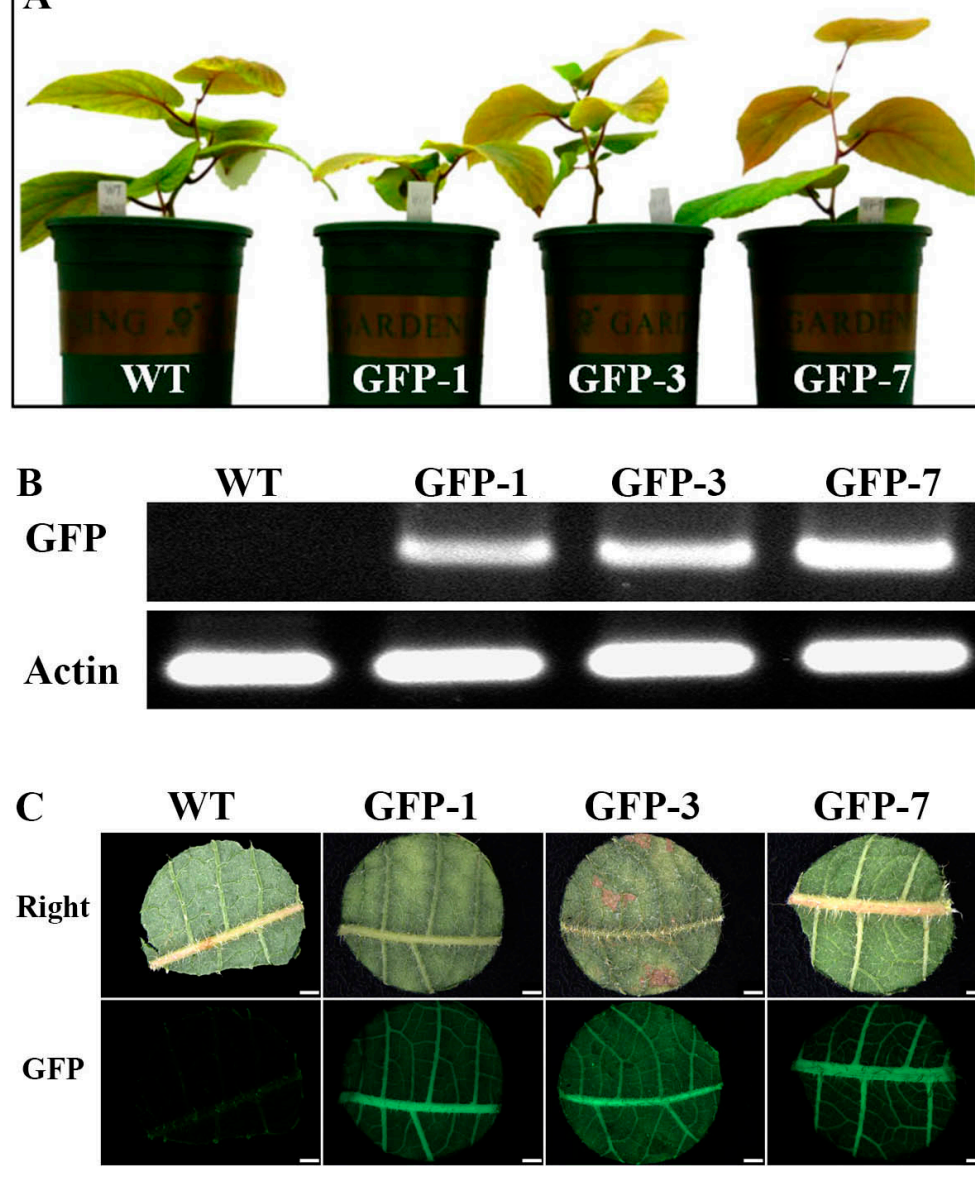
GFP observation from WT and three transgenic kiwifruits grown in greenhouse. (**A**) Three-month-old WT and three transgenic kiwifruits grown in greenhouse. (**B**) Detection of *GFP* expression from WT and three transgenic kiwifruits (**A**) by RT–PCR. (**C**) Observation of *GFP* expression from mature leaves of WT and three transgenic kiwifruits (**A**) using fluorescence microscopy. Bar = 1 mm.

To investigate how the multi-insertion of the *GFP* gene affects GFP expression in mature kiwifruit plants, DNA extracted from the mature leaves of two 2-copy insertion lines, GFP-1 and GFP-7; a 4-copy insertion line, GFP-3 (Figure 4); and a WT kiwifruit grown for 3 months in the greenhouse (Figure 5A) was analyzed for *GFP* expression with RT-PCR and fluorescence microscopy. The clear putative *GFP* band was amplified using RT-PCR analysis from three transgenic kiwifruit plants, while the band was not detected in the WT kiwifruit (Figure 5B). Moreover, strong *GFP* expression was detected in the leaves of the three transgenic lines, while *GFP* expression was not detected in the WT (Figure 5C), implying that the stable expression of *GFP* may not be affected by the number of GFP insertions in the transformed kiwifruit.

### 2.6. Procedure for Genetic Transformation of Kiwifruit

**Figure 6:**
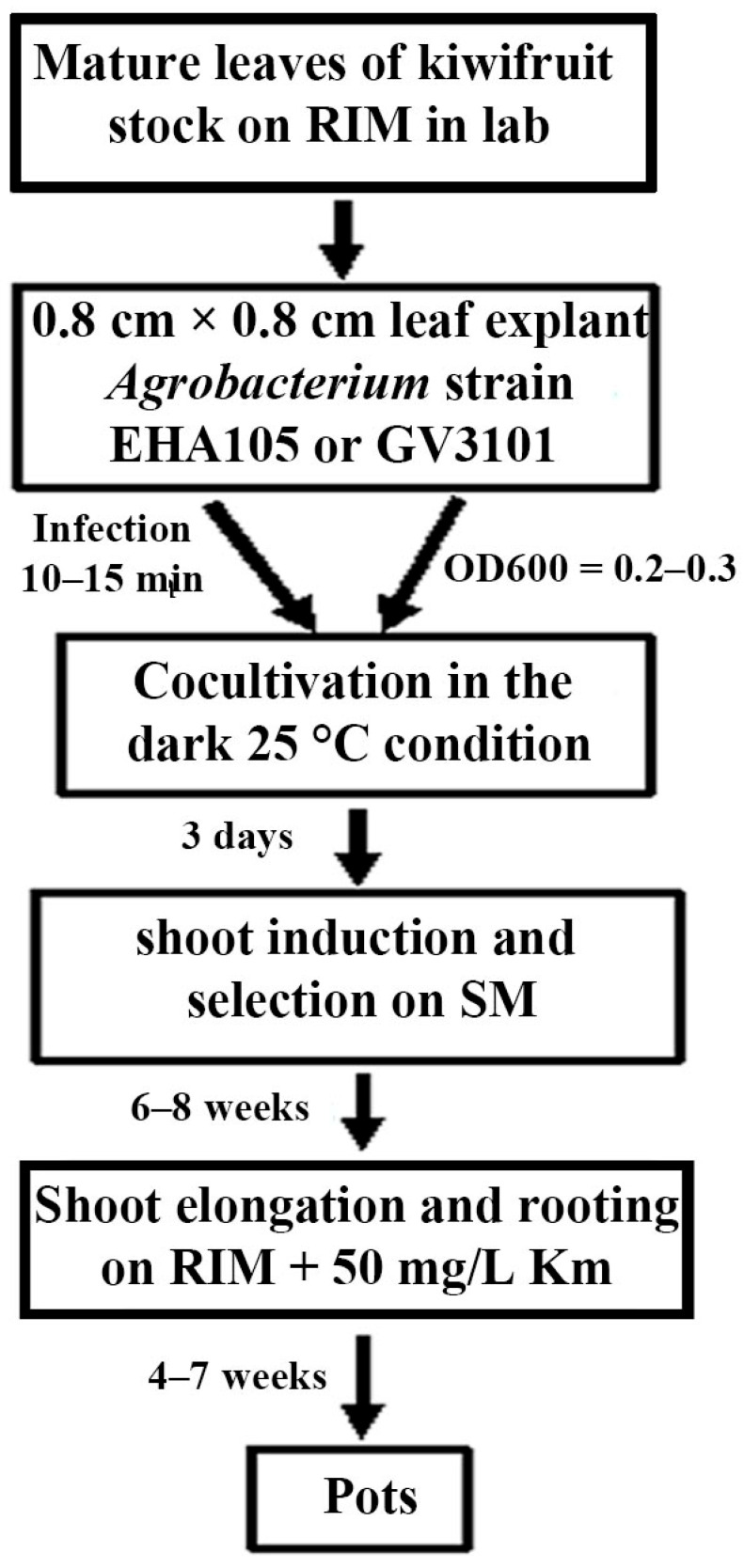
Summary of procedure for *Agrobacterium*-mediated genetic transformation of kiwifruit. Timetable is displayed on panel.

The procedure of plant regeneration and the *Agrobacterium*-mediated genetic transformation of kiwifruit is summarized in Figure 6. In the first step, leaf explants (0.8 cm × 0.8 cm) were infected with *Agrobacterium* strain EHA105 or GV3101 for 10–15 min. After three days of cocultivation on CM at 25 °C in the dark, the leaf explants were transferred to shoot induction and selection medium. After six to eight weeks of culture, kanamycin-resistant small adventitious shoots were transferred to shoot elongation and rooting medium. After four to seven weeks of culture, rooted plantlets exhibiting both kanamycin resistance and strong GFP expression were cultivated in pots or in soil. This genetic transformation procedure for kiwifruit required approximately 4 months.

## 3. Discussion

The tissue culture technique is an essential tool for the micropropagation and genetic transformation of plants [22,33,34]. The success of tissue culture is dependent on the genotype and explant types of the material, the composition of the culture medium, and the combination of plant hormones [35,36,37]. In kiwifruit, several studies have reported successful shoot regeneration and genetic transformation from hypocotyls, stems, and leaves [23,28,30]. For example, a combination of MS medium + 1.0 mg/l 4-PU (N-(2-chloro-4-pyrldyl)-N-phenylurea) or B5 medium + 3.0 mg/l zeatin from the hypocotyl and stems was used, and MS medium + 5 mg/L Zt + 0.1 mg/L NAA regenerated shoots from leaf explants via the callus phase [23,28]. Moreover, Zhang et al. (2018) [30] reported shoot regeneration from leaf explants using a combination of MS medium + Nitsch & Nitsch vitamins + 4 mg/L BA + 1 mg/L NAA. However, these methods have several limitations such as the induction and cultivation of calluses, low-efficiency shoot regeneration, and eventually successful genetic transformation. This requires a lengthy tissue culture period and labor-intensive processes. In the present study, modified 3/4 MS components were used as a basal medium, and the mature leaves of in vitro-grown kiwifruit as a source of explants were investigated for shoot regeneration frequency by treatment with several combinations of the cytokine BA with 1 mg/L Zt, a highly effective regulator for the induction of shoot morphogenesis [38,39]. Interestingly, we found that more than 93.3% of the leaf explants responded to 3/4 MS + 5 mg/L BA + 1 mg/L Zt + 0.15 mg/L IBA medium, and multi-adventitious shoot formation was stimulated from each leaf explant after 6 weeks of culture (Figure 1A), clearly greater compared to any other treatment combination (Table S2). This result implies that Zt is an important plant regulator and that 5 mg/L BA + 1 mg/L Zt is a suitable combination for shoot regeneration from kiwifruit leaf explants (Figure 1; Table S2). Furthermore, on this medium we successfully achieved stable genetic transformation and obtained several kanamycin-resistant and *GFP*-expressing shoots after 4 months of culture (Figure 2 and Figure 6). This transformation method requires shorter culture periods than other previously reported transformation methods [23,28,30]. Therefore, we suggest that leaves represent a better explant and that combination with modified 3/4 MS-based SIM is an efficient and suitable platform for micropropagation and the genetic transformation of kiwifruit cultivar ‘Hayward’.

The *Agrobacterium* strain is another important factor for successful genetic transformation in plants [37,40]. In this study, two common, strong *Agrobacterium* strains, EHA105/pBI-35S::GFP and GV3101/pBI-35S::GFP, were chosen as the T-DNA delivery system, and the transformation efficiency of these strains in kiwifruit was compared. We found that approximately 71.1% kanamycin-resistant plantlets of EHA105/pBI-35S::GFP and 72.5% kanamycin-resistant plantlets of GV3101/pBI-35S::GFP presented strong *GFP* expression 15 weeks after inoculation (Table 1; Figure 3A) and were stably expressed in three-month-old plants grown in the greenhouse (Figure 5). Moreover, *GFP* integration in the genome of kiwifruit was detected with Southern blot and PCR analysis, and expression was detected using RT-PCR (Figure 4 and Figure 5B). These findings imply that any strain of *Agrobacterium* EHA105 and GV3101 could be used to transform kiwifruit.

The *Agrobacterium*-mediated transformation system is an important deliverer for CRISPR-Cas9 genome editing in plants, with its efficiency significantly influencing genetic modification outcomes [22]). In this study, we employed this system to target the *PDS* gene in kiwifruit. An analysis of six hygromycin-resistant shoots confirmed the successful integration of the CRISPR-Cas9 editing cassette (Figure S2A,B), and a regenerated shoot displayed an albino phenotype (Figure S2C). Although sequencing validation was not performed, these results indicate that the improved transformation method could be valuable for functional gene studies and genome editing in kiwifruit.

In the present study, we described a reliable and efficient *Agrobacterium*-mediated kiwifruit transformation protocol using a combination of mature leaf explants and two common *Agrobacterium* strains, EHA105 and GV3101. The method requires a tissue culture period of approximately 4 months, after which over 71% of kanamycin-resistant transformed plantlets presented strong *GFP* expression. Moreover, a strong expression of *GFP* was detected in the mature leaves of three-month-old potted plants. Therefore, this protocol provides a reliable tool for the fundamental study of gene function and can be applied to produce improved new kiwifruit cultivars through biotechnique approaches, such as RNAi and genome editing

## 4. Materials and Methods

### 4.1. Plant Materials and Growth Conditions

This study used in vitro-grown ‘Hayward’ kiwifruit (*Actinidia deliciosa*) plants maintained at Zhejiang A&F University. The plants were cultured on solid RIM (Table S1) at 25 °C under a 16/8 h light/dark photoperiod with cool white fluorescent light (100 µmol m^−2^ s^−1^). Shoot tips (2–3 cm) were subcultured on fresh RIM every two months. Fully expanded mature leaves were used for transformation experiments and served as the wild-type (WT) control in molecular and *GFP* expression analysis.

### 4.2. Tissue Culture of Leaf Explants

To determine the optimal shoot regeneration conditions from leaves, leaf explants (0.8 cm × 0.8 cm) were excised from plantlets and cultured on ¾-strength MS basal medium [41] supplemented with varying concentrations and combinations of cytokinins (BA and Zt) along with 0.15 mg/L NAA (Table S2). Thirty explants were placed per treatment. After three weeks of cultivation, enlarged explants were subdivided and transferred to three identical fresh media for further culture. Cultures were maintained at 25 °C under a 16/8 h light/dark photoperiod with cool white fluorescent light (100 µmol m^−2^ s^−1^). Shoot regeneration frequency was evaluated after five weeks of cultivation.

### 4.3. Infection of Kiwifruit Leaf Explants

The phytohormones and antibiotics were filter-sterilized (0.22 μm) and aseptically added to the autoclaved culture medium (121 °C, 20 min) after cooling to approximately 70 °C before experimental use.

The kiwifruit transformation was conducted using two *A. tumefaciens* strains, EHA105/pBI-35S::GFP and GV3101/pBI-35S::GFP, both harboring the binary vector pBI-35S::GFP. This vector contained the neomycin phosphotransferase II (*NPT II*) gene as a kanamycin selectable marker and the green fluorescent protein (*GFP*) gene as a visible marker [42]. The *Agrobacterium* strains were cultured in 4 mL of liquid YEP medium (Table S1) supplemented with 50 mg/L kanamycin and 25 mg/L rifampicin, at 28 °C shaking at 200 rpm for 24 h. The bacterial suspension was then diluted 1:10 (OD_600_ = 0.2–0.3) with liquid IM medium (Table S1). Fresh kiwifruit leaves were cut into 0.8 cm × 0.8 cm segments, and 30 leaf explants per set were inoculated in the diluted *Agrobacterium* suspension for 15 min. After inoculation, the explants were cocultured on CM medium (Table S1) at 25 °C in the dark for 3 days. The experiment was performed twice. Following cocultivation, the leaf explants were transferred to SM (Table S1) for kanamycin-resistant shoot induction, with fresh SM replenished every two weeks. After two to three rounds of selection, shoots displaying *GFP* expression and normal growth were excised and rooted on RIM (Table S1) supplemented with 50 mg/L kanamycin and 300 mg/L cefotaxime. After 6 weeks of culture, rooted plantlets were individually transferred to 400 mL square plastic bottles containing RIM (Table S1) supplemented with 50 mg/L km and 300 mg/L cefotaxime to maintain them and for further molecular analysis and validations of *GFP* expression. Furthermore, small transformed plants identified by Southern blot analysis were propagated. Selected plants were then transferred to soil and assessed for *GFP* expression and morphological characteristics.

### 4.4. Observation of Visible GFP Expression

*GFP* expression was visualized using a fluorescence microscope (Leica 165 FC, Leica Microsystems, Mannheim, Germany) equipped with a GFP filter. Observations were performed at distinct developmental stages: in the emerging solid callus stage after 10 days post-infection, beginning at the adventitious shoot emergence stage after 3 weeks post-infection, at the elongation of shoot stage after 5 weeks post-infection, at the rooted small plant stage, and at the stage when mature leaves appear in 3-month-old greenhouse-grown plants. Images were captured using a Leica DFC310 FX CCD camera coupled to the microscope system.

### 4.5. PCR Detection of Transformed Kiwifruit Plants

Genomic DNA was extracted from the mature leaves of putatively transformed kiwifruit plants using a cetyltrimethylammonium bromide (CTAB)-based method [43]. The primer sets (Table S3) were used to amplify the *GFP* and *NPT II* genes. PCR amplification reactions were performed on a total volume of 20 µL containing 50 ng of DNA, a 200 µM dNTP mixture, 0.2 µM of each primer, 1 × FastPfu DNA polymerase reaction buffer, and 1 U FastPfu DNA polymerase (TransGen, Beijing, China) under denaturation at 96 °C for 3 min followed by 35 cycles of 96 °C for 30 s, 62 °C for 30 s, and 72 °C for 50 s, with a final extension at 72 °C for 10 min. The amplified product was analyzed with electrophoresis on 1% agarose gels at 100 V for 30 min.

### 4.6. Southern Blot Analysis

Genomic DNA was extracted from 1 g of fresh mature leaves collected from the pot-grown wild-type (WT) and seven transformed kiwifruit plants using a CTAB-based method [43]. Each 20 µg DNA sample was digested with *Bam*HI, electrophoresed on 0.8% agarose gels at 200 V for 10 h, and then transferred onto Hybond-N+ (Amersham) nylon membranes. A digoxin-labeled *GFP* probe was synthesized with PCR using the PCR Dig Probe Synthesis Kit (Roche, Basel, Switzerland). The hybridization and signal detection procedures were performed according to the manufacturer’s instructions (Roche).

### 4.7. Semiquantitative RT–PCR Analysis

Total RNA was isolated from the mature leaves of a wild-type plant and GFP-positive plants growing in the greenhouse using an EasyPure^®^ Plant RNA Kit (TransGen, Beijing, China). cDNA was synthesized with *TransScript*^®^ First-Strand cDNA Synthesis Super Mix (TransGen, Beijing, China). RT–PCR was carried out using the GFP-specific primer set described above, and the SnActin-specific primer set was used as a positive control (Table S3). The reaction was performed with 1 μL of the first cDNA sample as a template and started with denaturation at 98 °C for 2 min, followed by 30 cycles of 96 °C for 30 s, 62 °C for 30 s, and 72 °C for 50 min, with a final extension at 72 °C for 10 min. The amplified DNA fragments were separated with electrophoresis on 1.2% agarose gels at 100 V for 30 min, stained with ethidium bromide, and observed under UV illumination.

### 4.8. Construction of pHEE401E-AcPDS Vector

Two target sequences AcPDS-gRNA1 and AcPDS-gRNA2 (Table S3) were selected within the coding region of the *Actinidia phytoene desaturase* gene (AcPDS, XM_057618388.1) using the website http://www.crisprscan.org. Based on the pHEE401E backbone [44], the pHEE401E-AcPDS editing vector was constructed. The vector was introduced into *A. tumefaciens* strain GV3101 via electroporation for subsequent transformation assays.

## Figures and Tables

**Table 1:** Comparison of transformation efficiency of two common *Agrobacterium* strains EHA105/pBI-35S::GFP and GV3101/pBI-35S::GFP.

*A. tumefaciens* Strains (Plasmid)	Replicate	No. of Leaf Explants ^a^	Km^+^ Shoot Numbers ^b^	GFP-Positive Shoot		Transformation Frequency (%) ^d^
				Numbers	Frequency (%) ^c^	
EHA105 (pBI-35S::GFP)	1	30	24	17	70.8	80
	2	30	21	15	71.4	70
	Total	60	45	32	71.1	75
GV3101 (pBI-35S::GFP)	1	30	21	15	71.4	70
	2	30	19	14	73.7	63.3
	Total	60	40	29	72.5	66.7

^a^ Kiwifruit plant was maintained on RIM in a tissue culture room, and approximately 0.8 cm × 0.8 cm leaf explants were infected with *Agrobacterium* strains. ^b^ A single normally developed kanamycin-resistant (Km^+^) shoot was excised from a leaf explant after 8 weeks of cultivation on selection medium (SM) and transferred to RIM supplemented with 50 mg/L kanamycin and 300 mg/L cefotaxime. ^c^ (No. of GFP-positive shoots / no. of kanamycin-resistant shoots) × 100%. ^d^ (No. of kanamycin-resistant shoots / no. of leaf explants) × 100%.

## Data Availability

All data generated or analyzed during this study are included in the article and its Supplementary Materials.

## Supplementary Materials

The following supporting information can be downloaded at https://www.mdpi.com/article/10.3390/plants14152353/s1, Supplementary Table S1: Composition of medium used in this study. Supplementary Table S2: Effect of various concentrations and combinations of BA, Zt, and 0.15 mg/L IBA (in 3/4MS basal medium) on shoot regeneration from leaf explants of kiwifruit. Results were scored 5 weeks after culture. Supplementary Table S3: The primers used for the detection of transformed plants, RT-PCR, and the genome editing analysis in this study. Supplementary Figure S1: Detection of kanamycin-resistant *NPT II* gene in transformed kiwifruits. Supplementary Figure S2: An insertion analysis of the editing cassette and PCR detection of the *hygromycin-B* gene.
