# Supplementary material for: An Improved Agrobacterium-Mediated Transformation Method for an Important Fresh Fruit: Kiwifruit (Actinidia deliciosa)
Source: Plants (Basel). 2025 Jul 31;14(15):2353. doi: 10.3390/plants14152353 (PMC12349596; doi:10.3390/plants14152353)
Supplement: Supplementary file 1 [file plants-14-02353-s001.zip › plants-3734297-supplementary.pdf]

Supplementary Materials

Improved *Agrobacterium*-Mediated Transformation Method for an Important Fresh Fruit Kiwifruit (*Actinidia deliciosa*)

Chun-Lan Piao[1†], Mengdou Ding[1, 2†], Yongbin Gao[1†], Tao Song[2], Ying Zhu[2*], Min-Long Cui[1*]

[1] Key Laboratory of Quality and Safety Control for Subtropical Fruit and Vegetable, Ministry of Agriculture and Rural Affairs, College of Horticulture Science, Zhejiang A&F University, Hangzhou 311300, China.

[2] State Key Laboratory for Managing Biotic and Chemical Threats to the Quality and Safety of Agro-Products, Key Laboratory of Traceability for Agricultural Genetically Modified Organisms, Ministry of Agriculture and Rural Affairs, Institute of Virology and Biotechnology, Zhejiang Academy of Agricultural Sciences, Hangzhou 310021, China

† These authors contributed equally to this work.

*** Correspondence:** Min-Long Cui; Ying Zhu

Min-Long Cui: minlong.cui@zafu.edu.cn; Tel: +86-(571)63611387

Ying Zhu: yzhuzaas@163.com;

Supplementary Table S1: Composition of media used in this study

**YEP medium:**	10 g/L peptone + 5 g/L NaCl +10 g/L yeast extract + pH 7.0
**Shoot Induction medium (SIM):**	3/4 MS + 30 g/L sucrose + 5 mg/L BA + 1 mg/L Zt + 0.15 mg/L IBA + 8 g/L Agar + pH 5.8
**Root Induction medium (RIM):**	1/2MS + 25 g/L sucrose + 7 g/L Agar + 0.25 mg/L IBA + pH 5.8
**Inoculation medium (IM):**	MS + 30 g/L sucrose + 2 mg/L BA + 0.15 mg/L NAA +100 mM AS + pH 5.2
**Cocultivation medium (CM):**	SIM + 100 mM AS + pH 5.8
**Selection medium (SM):**	SIM + 100 mg/L Km + 300 mg/L Cef

MS: Murashige and Skoog medium (M519, PhytoTech Labs, United States); BA: 6-Benzylamino purine (B3408, Sigma, Aldrich); Zt: Zeatin (A411990, SangonBiotech, China); NAA: 1-Naphthaleneacetic acid (N0640, Sigma, Aldrich); IBA: Indole-3-butyric acid (I5386, Sigma, Aldrich) ; AS: Acetosyringone (AB1111, SangonBiotech, China); Km: Kanamycin (A430277, SangonBiotech,China; Cef: Cefotaxime (A601276, Sangon Biotech, China); Sucrose (S2792, SangonBiotech, China); Agar (A1296, Sigma, Aldrich)

Supplementary table S2: Effect of various concentrations and combinations of BA, Zt and 0.15 mg/L IBA (in 3/4MS basal medium) on shoot regeneration from leaf explants of kiwifruit. The results were scored 5 weeks after culture.

6-BAP (mg.L-1)	Zt (mg.L-1)	IBA (mg.L-1)	No. of leaf explants	No. of shoot-forming explants (%) [a]	No. of independent shoots per explants [b-]
0.0	0.0	0.0	30	0 (0.0)	
1.0	0.0	0.15	30	0 (0.0)	
2.0	0.0	0.15	30	2 (6.7)	
3.0	0.0	0.15	30	3 (10.0)	
4.0	0.0	0.15	30	6 (20.0)	0.52 ± 1.15
5.0	0.0	0.15	30	12 (40.0)	1.26 ± 1.73
1.0	1.0	0.15	30	4 (13.3)	
2.0	1.0	0.15	30	9 (30.0)	1.17 ± 1.85
3.0	1.0	0.15	30	18 (60.0)	2.83 ± 2.49
4.0	1.0	0.15	30	25 (83.3)	4.52 ± 2.38
5.0	1.0	0.15	30	28 (93.3)	10.87 ± 3.71

[a] No. of shoot-formed leaf explants / No. of leaf explants × 100%

Supplementary Table S3: The primers used for detection of transformed plants and RT-PCR analysis in this study.

GFP-F: ATGGTGAGCAAGGGCGAGGAGC
GFP-R: TTACTTGTACAGCTCGTCCATG C
NPTII-F: AGATGGATTGCACGCAGGTTC
NPTII-R: GTGGTCGAATGGGCAG GTAG
SnActin-F: CGCGCGCTACACTGATGTATTCAA
SnActin-R: TACAA AGGGCAGGGACGTAGTCA A
AcPDS-gRNA1: CTTGGAGATGTGGTTGTCAC
AcPDS-gRNA2: TGGTCTTGGATAATCCATAC
U6-26F: TGTCCCAGGATTAGAATGATTAGGC
U6-26R: AGCCCTCTTCTTTCGATCCATCAAC
Hyg-F: ACCTGCCTGAAACCGAACT
Hyg-R: GCTGCTCCATACAAGCCAA
AcPDS-F: ATGTTGCATATTGGGTTTGAT
AcPDS-R: TCAGCAATAGAGAGATATGTT

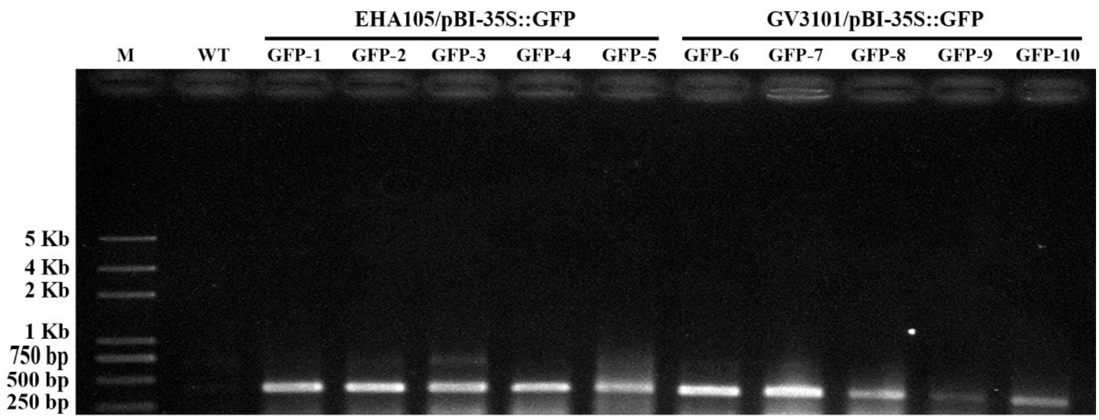

Supplementary Figure S1. Detection of the kanamycin-resistant *NPT II* gene in transformed kiwifruits. Genomic DNA was extracted from fresh leaves of pot-grown wild-type (WT) and ten putative transgenic GFP-positive kiwifruit lines. PCR analysis was conducted using *NPT II*-specific primers (Table S3). WT**:** Non-transformed wild-type kiwifruit. GFP1–5**:** Five independent GFP-positive transgenic lines transformed with EHA105/pBI-35S::GFP. GFP6–10**:** Five independent GFP-positive transgenic lines transformed with GV3101/pBI-35S::GFP. M: D2000 Plus DNA Marker (Takara, Japan).

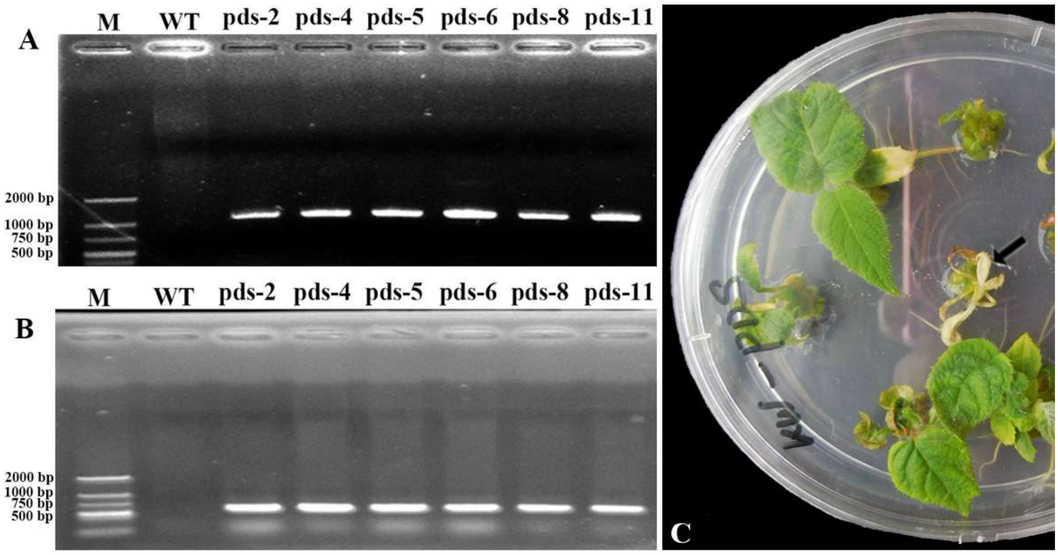

Supplementary Figure S2. Insertion analysis of the editing cassette and PCR detection of the *hygromycin-B* gene. Genomic DNA was extracted from fresh leaves of wild-type (WT) plants grown in the plastic pot and six hygromycin-resistant masses transformed with GV3101/pHEE401E-AcPDS. (A) PCR amplification of the editing cassette using U6-26F/U6-26R primers (Table S3). (B) PCR amplification of the hygromycin-B gene using Hyg-F/Hyg-R primers (Table S3). M: DL2000 DNA marker; WT: negative control; pds2, 4-6, 8, and 11: six independent hygromycin-resistant kiwifruit masses. (C) Phenotypic analysis of hygromycin-resistant T_0 plantlets on rooting medium after 6 weeks of culture. An albino plantlet is indicated by a black arrow.
